# Legislative Intent and Agency: A Rational Unity Account

**DOI:** 10.1093/ojls/gqae001

**Published:** 2024-02-09

**Authors:** Stephanie Collins, David Tan

**Affiliations:** Associate Professor of Philosophy, Monash University

**Keywords:** legislative intent, intentionalism, group agency, legal interpretation

## Abstract

Realist theories of legislative intent can be divided between aggregative theories (on which legislative intent is what some proportion of legislators intend) and common intent theories (on which legislative intent is a unanimous intent among legislators). In this article, we advance and defend an alternative realist conception of legislative intent: the rational unity account. On this account, the legislature is an agent with a distinctive ‘rational point of view’—a concept we adopt from social ontology. The legislature’s rational point of view is shaped by its procedures and structures, in ways not determined by either a common intention held by legislators or an aggregation of the intentions of legislators. We explain how our view improves on existing accounts. We then apply it to three cases to demonstrate its implications for legal interpretation. Importantly, on the proposed account, legislative intent can depart from what individual legislators think or know.

## 1. Introduction

When considering the intention behind a statute, ambivalence is natural. On the one hand, the legislature is a radically diverse group: why think that opponents on different sides of the aisle (or within one party) share a unitary intention? On the other hand, it cannot be that statutes are created for no reason: legislative intent seems necessary for understanding law making as a rational action.^1^

This article develops a new theory of legislative intent that captures both sides of this ambivalence. The article proceeds in four parts. In section 2, we provide an overview of the debate about legislative intent. We propose three sides to this debate: scepticism, realism and constructivism. We describe four hurdles that any realist account of legislative intent must overcome and explain how existing versions of realism have difficulties overcoming them.

In sections 3 and 4, we present a new realist account of legislative intent—the *rational unity account*—which improves upon prevailing ‘aggregative’ and ‘common intent’ versions of realism. On the account, the legislature is an agent with a distinctive ‘rational point of view’ (RPOV)—a concept we adopt from social ontology. The legislature’s RPOV is not determined by either an aggregation of legislators’ intentions (as on aggregative views) or a unanimous intention held by legislators (as on common intent views).^2^ Our account encourages proponents of legislative intent to look beyond the thoughts of the people who happen to sit in the chamber when a bill passes into law, and to the rational point of view of the legislature across time. Under our proposal, the intention behind a particular statute is determined by how that statute relates to all other propositions to which the legislature is committed, where these commitments are not dependent on the thoughts or actions of legislators when the bill passes. We show how this account overcomes the above-mentioned hurdles. Section 5 applies this account to statutory interpretation, demonstrating how it helps overcome the epistemic barriers to accessing the legislature’s intentions. Section 6 concludes.

Throughout, we employ a distinction between a legislator, the legislators and the legislature. A legislator is a human individual who occupies a role as lawmaker. ‘The legislators’ refers to a collection of individuals who are all legislators in the same jurisdiction and at the same time. In any democratic jurisdiction at any time, the legislators are a diverse plurality, each of whom has their own intentions, goals, plans and so on. The legislature, in our account, is not identified with the collection that is the legislators. Unlike the legislators, the legislature has a single unified set of intentions, goals, plans and so on. The legislature survives changes in the legislators (as when a member resigns or is voted out). It is an open question whether the legislature, in this sense, even exists. We provide the theoretical tools for an affirmative answer.^3^

This article primarily draws on contemporary theories in social ontology (a philosophical subdiscipline that theorises social facts and institutions) and, more specifically, theories of group agency (theories that model how groups can be agents). To illustrate the theoretical elements of the article, we will mainly use the Australian legislature as an example. There is much existing work on legislative intent in the US and UK legislatures; hence, using Australia as an example provides novelty. Nonetheless, the examples we provide are generalisable to other jurisdictions.

There is a further question, not addressed by this article: if legislative intent exists, is it relevant to legal interpretation? One might be a realist about legislative intent, while, for normative reasons, thinking it irrelevant to the correct interpretation of a statute. We will not deal with this normative question here. We address only the underlying metaphysical question and some of the epistemic issues. But our argument is normatively important. If the metaphysical and epistemic questions cannot be satisfactorily answered, then the normative question is moot: if there is no such thing as legislative intent, or if that intent can never be accessed, then legislative intent can hardly guide the interpretation of statute.

## 2. The Debate over Legislative Intent

Views on legislative intent in legal scholarship can be divided into three camps: *sceptics*,^4^*constructivists*^5^ and *realists*.^6^

Sceptics either do not think legislative intent exists (metaphysical scepticism) or they believe that if it does exist it can never be discovered (epistemic scepticism). As Shepsle characterises metaphysical scepticism: ‘legislative intent is an internally inconsistent, self-contradictory expression’.^7^ Constructivists believe legislative intent exists, but believe it is not fully dependent on facts about legislators or the legislature; instead, legislative intent is partially *constructed* by practices outside the legislature.^8^ Thus, metaphysical sceptics believe the term ‘legislative intention’ has no referent, just as ‘ether’ and ‘ghosts’ have no referents. Constructivists believe ‘legislative intention’ has a referent, but do not understand that referent as realists do.

Realists posit that legislative intentions are *fully dependent* on facts about legislators and/or the legislature and can at least sometimes be *discovered* (not co-created) by those outside the legislature. For realists, the intention behind a statute is settled by the time the legislation passes. Later interpretations (by judges, civil servants or others) have no effect on the content of the intention. Such audiences *discover* the legislative intent, rather than the interpretive process being a *constitutive element* of the true intentional content.

Realist views can be divided into theories that emphasise *common intent* and those that emphasise *aggregation* or *social choice*. The former posit a unanimous intent of legislators.^9^ The latter acknowledge disagreements between legislators, which are resolved through an aggregative function (eg majority choice).^10^

Both realist theories face the following obstacles:

No-Cooperation: Legislators do not agree, coordinate or cooperate on the meaning of the text.^11^

Ignorance: Legislators often are not aware of the full text of bills or have not thought about what the text means.^12^

Insincerity: The true preferences or intentions of legislators may be hidden from external observers due to legislative compromises and strategic voting.^13^

Grouphood: There must be a reason to attribute the intention to the *legislature* rather than to an aggregate of individuals.^14^

Aggregative theories deal well with No-Cooperation, but not Ignorance, Insincerity or Grouphood. That is, we can easily aggregate the intentions of legislators even if they disagree or lack cooperation: the whole purpose of aggregation in social choice theory is to deal with situations involving disagreement. Aggregation confronts problems concerning cooperation only when the aggregation rule breaks down. For example, there are well-known circumstances under which no aggregated intent can be determined.^15^ However, there is also plenty of work both generally^16^ and in law^17^ that aims to resolve these problems.

Concerning Ignorance, aggregation faces difficulties. It is impossible to aggregate views within a group if some members have no view. Indeed, it is often drafters and a small group of legislators who construct statutes; other legislators might have no views to aggregate. Regarding Insincerity, the possibility of compromises and strategic decision making make it difficult to ascertain what individuals believe about a text, which makes it difficult for outsiders to aggregate those beliefs. Finally, Grouphood poses problems for aggregation. The legislature’s rules do not stipulate that the legislature’s intention is determined by aggregating members’ intentions, providing no reason to view members’ aggregated intentions as more than a statistical summary of individuals’ intentions.^18^

Common intent theories also face the hurdles. A *proposition-wise* common intent theory requires all legislators to intend a proposition if the legislature is to intend that proposition. Such theories flounder at No-Cooperation, since it is rare that every legislator will have the same intention for any given statute—or even for the goals of the legislature overall. Concerning Ignorance, if not all legislators have thought about a proposition, then there can be no common intent regarding it. Likewise for Insincerity: if compromises and strategic voting hide legislators’ intentions from observers, then we cannot know whether legislators have common intent.

Common intent theories also have trouble with Grouphood. If all individuals in a body intend something, this does not imply *the body* intends it. To see this, consider that all members of a university might hold a particular political view without it being true that *the university*—or even *the faculty*—holds that political view. The university holds that political view only if the view has been ratified by the university’s procedures and structures. We explain this further in section 3B.^19^

Nonetheless, Richard Ekins—perhaps the best-known common intent theorist—is not a *proposition-wise* common intent theorist. In Ekins’s account, the content of legislative intent is an ‘open proposal’ that is ‘capable of being known’ by all legislators.^20^ An ‘open proposal’ is defined as ‘the meaning that a reasonable sole legislator who attends to the context—would be likely to intend’.^21^ The legislators need not *unanimously intend* the meaning of that hypothetical sole legislator. Instead, common intent enters Ekins’s account when he posits legislators’ unanimous intent to *create laws for the common good*. This purported common intent faces all four hurdles: there still might not be agreement on this intent to create laws for the common good, or at least legislators might not have thought about this intent; this intent might be obscured to outsiders; and some positive account is required of why this intent should be attributed to *the legislature*.^22^

Further, these two elements of Ekins’s theory—first, open proposals, and second, common intent to create law for the common good—are independent. This article is not antagonistic to Ekins’s ‘open proposals’, considered independently of common intent. Yet at least two aspects of the open proposals are underdeveloped. First, it is unclear how open proposals relate to Ekins’s conception of the legislature’s agency. The meaning that would be intended by a hypothetical sole legislator seems disconnected from the legislature itself.^23^ Second, *what* the reasonable sole legislator would intend remains ill-defined. What forms of reasoning are employed by the sole legislator? How can real-world statutory interpreters glean the intentions of this hypothetical person? Those sympathetic to Ekins might use some of our theoretical resources to complement and fill these gaps in his account (see section 4C).

## 3. The Rational Unity Account

### A. Rationally Unified Agents

The rational unity account (RUA) adapts recent philosophical work in social ontology.^24^ To begin, consider a foundational question: what is an agent? Definitionally, an agent is an entity that acts. To act is to perform behaviour that is intentional under some description.^25^ Thus, all agents have intentions. What, then, is an intention? Social ontologists often adhere to *functionalism* about mental states, where ‘mental states’ encompass intentions, beliefs, preferences, regrets, fears, wishes and so on. On functionalism, mental states are characterised by their relationship to other mental states, to behavioural dispositions and perhaps to other properties of the agent.^26^

For example, if I *intend* to eat cake, then I am *disposed* put cake in my mouth, I *believe* it is possible for me to eat cake and I *prefer* eating cake over not. These other states are the ‘function’ of my intention. On functionalism, my intention to eat cake is constituted by such dispositions, beliefs and preferences; which, in turn, are constituted in part by other mental states and dispositions. Further, philosophers of mind generally agree on the following: if I intend to eat cake, then I must have some beliefs (perhaps tacitly) about cake. These beliefs, in turn, require other mental states that are not about cake per se. For example, if I believe cake is food, then I must have some belief about what ‘food’ is. Likewise, if I believe food is sustenance, then I must have some belief about what ‘sustenance’ is, and so on for a range of inter-defined concepts.^27^ Under functionalism, an agent is any entity that has enough inter-defined mental states (which are characterised by those states’ functions) to provide meaningful content to the entity’s intentions (which are also characterised by their functions).

To have a meaningful intention—and, therefore, to be an agent—an entity must have a fairly large web of other mental states and behavioural dispositions. The web must be large enough to give some more-or-less determinate role and content to the intention (where the ‘role’ of a mental state is the way that mental state interacts with the agent’s other mental states, behavioural dispositions and so on). These ‘mental states’ need not reside in brains, since mental states are defined functionally. A mental state is not defined biologically (in terms of what physical substrate underlies the mental state), but rather by the state’s relation to other dispositions or properties. We follow Carol Rovane in using ‘rational point of view’ (RPOV) to refer to the interdependent web of mental states and action dispositions that an agent holds.^28^ A large RPOV is necessary if the agent’s intention is to have sufficient determinacy for interpretation and application by outsiders.^29^

If an agent is *rational*, then it operates to increase the *coherence* within its RPOV, at least sometimes and to some extent. A fully coherent RPOV contains no inconsistencies. For example, a fully rational agent would not hold an all-things-considered intention to eat cake *and* an all-things-considered intention not to eat cake. Moreover, a fully coherent RPOV contains relations of justificatory support between items in the RPOV (where ‘items’ includes mental states and behavioural dispositions). Clearly, no agent ever achieves full coherence. A rational agent has a tendency to bring its RPOV towards coherence and act on that basis, at least on occasion and to an extent.^30^ We do not here specify *how* robust this tendency must be for an entity to count as ‘rational’. If the standard is set too high, then no human adult would count as rational. Suffice to say, there are degrees of rational agency. Both individual humans and social entities can demonstrate some degree of rationality.

The core thesis of the RUA is that an agent is picked out by their RPOV, rather than by their body or brain.^31^ This approach to agency is general: it aims to capture *any* agent (including humans). Individual humans are not privileged as the locus of agency. Because the focus of the account is a bundle of interdependent mental states and dispositions, this can also include social or corporate entities that have such a bundle.^32^

### B. Social Entities

How does a social entity—such as a corporation, charity, church, university or legislature—form an RPOV?^33^ And how does a social entity increase coherence within its RPOV? Consider a university: a university’s political intentions are not determined by *aggregating* the political views of all faculty. Nor are they settled by *common intent*: all members of the university could have a common intent to bring down their federal government without the university having that intent. Instead, the university’s political intentions are determined in ways specified in the university’s procedures and structures. The details of the procedures and structures vary from university to university. Perhaps the university’s political intentions are determined by a vote in the university senate; by a committee that deliberates until a committee consensus is reached; by consultation with external stakeholders; by compromise among the holders of governance portfolios; or by decree from the vice-chancellor. The procedures and structures need not use aggregation or common intent.

The focus on procedures and structures should be extended to other social entities, for two reasons. First, procedures and structures enable a coherent set of commitments. Including all commitments of all individual members within a social entity’s RPOV would quickly lead to incoherence. While agents need not be maximally coherent, they should have a tendency to correct for incoherencies. Procedures and structures enable this. Secondly, there is consensus among social ontologists that a social entity is a *distinct agent*. Given this, it is not necessary to know the mental states of members, but to identify the social entity’s *distinctive* commitments. Procedures and structures enable this.

This is reflected in prominent theories of social entities’ agency. In Peter French’s influential theory of corporations’ agency, procedures and structures are captured by the ‘Corporate Internal Decision structure’, or CID structure.^34^ The CID structure is the centrepiece of French’s theory. It is embodied in ‘(1) an organizational or responsibility flow chart that delineates stations and levels within the corporate power structure and (2) corporate decision recognition rule(s) (usually embedded in something called “corporation policy”)’.^35^ When an item (ie mental state or behavioural disposition) is duly authorised by the corporation’s recognition rules, it becomes attributable to the corporation. In the terminology we have introduced, it is included in the organisation’s RPOV. French does not require common intent among members or an aggregation of members’ attitudes. All the CID structure requires of a member is that they play their role as specified in the organisational chart. If enough members play their roles adequately, the CID structure will produce group-level beliefs, preferences and intentions.

Procedures and structures are likewise foregrounded in List and Pettit’s recently influential theory of group agency, which they apply to courts, corporations, states and other social entities.^36^ They outline the problems with determining a group’s attitude towards a proposition by taking a majority vote of members on that proposition. The problem, roughly, is that proposition-by-proposition voting enables the group to form *contradictory views* on different propositions that are related to each other.^37^ In our terminology, proposition-by-proposition voting prevents the group from having a tendency towards coherence within its RPOV. List and Pettit therefore recommend that groups adopt *procedures and structures* that allow the group’s attitude towards proposition P to be determined by the group’s attitude towards propositions related to P, rather than by members’ attitudes towards P.^38^ Thus, procedures and structures are highlighted in List and Pettit’s theory of social entities’ agency.

Rovane, French, and List and Pettit are not the only philosophers whose accounts of social entities’ agency both (i) accord with the RUA and (ii) privilege procedures and structures. Raimo Tuomela analyses a group agent as ‘an interactive social system … that consists of interrelated individuals such that this system is, through them, capable of producing uniform actions and outcomes’.^39^ Members must be *integrated into a system* for group-level intentions to emerge: aggregated or shared intent is insufficient for group intent. Similarly, Kendy Hess uses Rovane’s account of agency to argue that corporations have their own RPOVs and, therefore, their own agency and intentions.^40^ Hess argues that corporations’ agency, RPOVs and intentions do not require shared intentions amongst members^41^ nor aggregation of members’ attitudes.^42^ Likewise, Deborah Tollefsen argues that a group is an agent when it is robustly explained and predicted by attributing to the group a ‘unified perspective—a rational point of view—and … norms of rationality’.^43^ She notes that aggregation and common intent are just two of many mechanisms that might underly such group agency.^44^ Finally, Tracy Isaacs views organisations as ‘structures in which the organizational roles and authority structures are outlined in the organization’s policy’.^45^ She states that ‘in order to understand the organisational intention there is no need to refer to the intentions of individuals. The content of their intentions is entirely beside the point’^46^—*contra* the aggregative and common intent accounts.

A rational social entity, then, need not aim at coherence among all the mental states and behavioural dispositions *of members*. On the RUA, each member has their own individual RPOV *plus* there is an additional RPOV that belongs to the social entity. The RPOV of one member can be completely different from the RPOV of another and completely different from the RPOV of the social entity—even while the social entity and all members are rational. This can happen because the social entity does *not* automatically hold all mental states and behavioural dispositions that any member holds, that a majority of members hold or even that all members hold. It holds the mental states determined by its procedures and structures. In the next section, we apply these ideas to the legislature.

## 4. The Rational Legislature

### A. The Legislature’s RPOV

Are there procedures and structures that enable the legislature to arrive at *its own* bundle of mental states and behavioural dispositions, where these are consistent enough and interlocking enough to enable the legislature to hold meaningful intentions in the functionalist sense we outlined above? We argue that there are. These procedures and structures are primarily laid out in legislatures’ *standing orders or rules*.^47^ However, not all structures and procedures of legislatures are explicit. There might be procedures beyond the standing orders, for example, norms or conventions of the legislature that are not formally written down. The legislature’s RPOV is determined by the actual structures and procedures the body uses to make decisions. These include, but are not necessarily limited to, the standing orders.

We take the Australian legislature as an example. Australia’s federal legislature is vested with the power to make legislation under section 1 of Australia’s Constitution. The process for passing legislation is laid out in the standing orders as follows for the lower house (the Australian Senate follows a similar procedure). First, a bill is read for the first time, with an explanatory memorandum stating reasons for the statute.^48^ Next, there is a second reading at which the relevant minister gives a speech and the bill is debated.^49^ Any proposed amendments are debated in the ‘Consideration-in-detail’.^50^ Finally, the bill is voted on at the third reading.^51^

These procedures and structures enable legislatures to pass reasoned legislation. It does not matter whether legislators endorse this process, have any intent in common, vote for reasons or at random, or think the debate is genuine.^52^ The point is that *the legislature* (not necessarily *the legislators*) has a commitment to reasoned legislation, encoded in the legislature’s procedures and structures.^53^ The procedures and structures require *reasons* to be provided with the bill, through the explanatory memorandum and the minister’s speech. Therefore, the legislature operates with a view to *coherently reasoned policy* for the statute.^54^

Note also that judicial views on the policy aspects of the bill do not enter the legislature’s RPOV. The procedures of the legislature (outlined above) demand behaviours of legislators alone, not judicial staff. Further, section 1 of the Constitution vests legislative power in the legislature, and the legislature alone—not judicial staff. This distinguishes the rational unity account from constructivist accounts.

The above implies that the following falls within the RPOV of the Australian legislature:

*Minimal RPOV:* The Australian legislature’s RPOV includes (i) explanatory memoranda,^55^ (ii) ministers’ second reading speeches, (iii) committee reports, (iv) the text of bills passed at the third reading, (v) other statutes and (vi) contextual general knowledge.

Items (v) and (vi) are not mentioned in the standing orders. Some explanation is needed for their inclusion. Other statutes are included for two reasons. First, the legislature does not rewrite legislation from scratch each time it sits or after each general election. Instead, all existing pieces of legislation in the statute books are commitments of the legislature. Under the RUA, social entities persist through changes in membership (in a legislature’s case, the entity persists through general elections). So, commitments made in earlier sittings form part of the legislature’s RPOV. Second, the standing orders encode a commitment to *reasoned* legislation. A statute is unreasonable if it gives instructions that contradict a previous statute.^56^ This provides an explanation, from the perspective of legislative intent, for typical legal doctrines for resolving statutory inconsistencies. Such doctrines include ‘harmonious construction’ (which requires judges to find interpretations that are consistent with supposedly conflicting provisions) and ‘implied repeal’ (which states that the more recent provision trumps the older one).^57^

Item (vi), contextual general knowledge, is best explained via an example. Suppose the legislature passes a bill about fishing. In interpreting this bill, we should attribute to the legislature generally known facts about fishing, even if no such information was brought up in the explanatory memorandum, second reading speech or committee reports. Such general knowledge is included in the RPOV because, as noted above, the standing orders encode a commitment to *reasoned* legislation. It would be impossible for a legislature to enact reasoned legislation about topic *X* if generally known facts about *X* were not assumed. These generally known facts give content to the legislation. By ‘generally known facts’, we do not mean facts that are known by every legislator: again, the legislators’ respective RPOVs are distinct from the RPOV of the legislature. Instead, as a rough guide, a proposition is ‘generally known’ when it is not open to dispute in the context of the bill. If a legislator were to introduce a generally known fact in parliamentary debate, they would be wasting the legislature’s time. Under this test, some fact could be ‘generally known’ even if not all legislators know the fact.^58^ Recall that, under our account, the procedures and structure of the parliament are independent of its members’ individual knowledge.

By including items (i)–(vi), a legislature’s RPOV is still rather thin. But (i)–(vi) are the *minimal* included items. More expansive conceptions of legislatures’ RPOVs are possible and should be a matter of debate among legal theorists who have expertise concerning the relevant legislature. Consider:

*Moderate RPOV:* all items (i)–(vi), plus (vii) legislative debates.

*Broad RPOV:* all items (i)–(vii), plus (viii) interpretive canons from common law.

The moderate RPOV might be adopted if and when debates are demanded by the standing orders of the legislature. However, this would often sacrifice coherence in the RPOV, since many contributions to debates will be inconsistent with one another. Therefore, only those debate contributions that do not introduce extreme inconsistencies should be included if one adopts the moderate RPOV. Meanwhile, the broad RPOV expands ‘generally known facts’ to include common law interpretive canons. Such canons are not as widely known as, say, general facts about fishing. Yet, these canons are known by legal practitioners and courts that interpret the bill, so knowledge of the canons might be taken as part of the reasonable law-making process.

We do not choose between the minimal, moderate and broad RPOVs. These are different positions that a theorist or judge may adopt, depending on how broad one thinks the structures and procedures of the legislature are. Just as theorists and judges might disagree about whether textualism or intentionalism is the correct approach to interpretation, so too they can also disagree about the appropriate inclusions in the RPOV of the specific legislature they are interpreting. We do not demonstrate which view is preferable, but merely aim to show that each is a defensible view of legislative agency and intent. Whichever RPOV one prefers, the RUA of legislative intent says that those applying a statute should use different evidence than common intent accounts or aggregative accounts—as we soon explain. Our goal in this article is to introduce and defend the RUA. Debates about the precise contours of specific legislatures’ RPOVs must await subsequent debate by theorists and judges.

That said, as a matter of rational unity, the same choice (between the narrow, moderate or broad approaches) should be made for all interpretations of a given legislature at a given period of time. It would lead to rational incoherence for a given legislature to possess both a broad and minimal RPOV at the same time (given the minimal approach is defined as excluding anything broader). It might be possible, however, for a legislature to change the contours of its RPOV over time. For example, suppose the standing rules were modified so that explanatory memoranda and second reading speeches always had to refer to common law interpretive canons. In such a situation, those canons should then be included in the RPOV. This would be no different from a human who changes how they form their attitudes at different stages of their lives, such that they should be interpreted differently. Legislative intent, however, is always determined by the RPOV at the time that the legislation was passed.

### B. Legal Content and Application

So far, we have presented a somewhat idealised model of parliamentary procedures, with reasoned debate adding coherent items into the RPOV at each stage of the process. The real world is not so simple; sometimes multiple committees are involved, with various amendments of bills being made by actors with conflicting motivations. These items might make it into the RPOV—even with the minimal interpretation of the RPOV. Further guidance is therefore required before interpreters can use our account.

To properly utilise our account amidst such complexity, one must distinguish the *content* of legislative intent from its *application*.^59^ This distinction is best illustrated via an analogy. Suppose I tell you that I intend ‘to be a philosopher’. The *content* of this mental state is a proposition about being a philosopher. I leave it to you to *apply* my will. For example, you must submit job or university applications on my behalf. I might have only a hazy idea of what I mean by ‘being a philosopher’. The content of my intention might be indeterminate regarding, for example, whether I intend to apply for philosophy PhD programmes or merely read philosophy in my spare time. Or perhaps I have many confused ideas about what being a philosopher entails. In applying my intention, you have only my intention ‘to be a philosopher’, combined with everything else you know about other items in my RPOV—which might not be much. In this respect, you are analogous to a judge who must apply legislative intentions. You cannot easily identify the *content* of my intention, perhaps because that content itself is indeterminate or because there too many conflicting pieces of evidence. Nonetheless, you must *apply* my intention. We use ‘statutory interpretation’ to include both content identification and application.

On the RUA, the *content* of legislative intent is metaphysically determined by all the other items in the RPOV. Thus, statutes are given their content through the RPOV with variations depending on whether the minimal, moderate or broad approach is correct. But once all these RPOV items are included and considered, judges might be left with indeterminacies or inconsistencies. How should these be overcome? Regardless of whether one advocates a minimal, moderate or broad account of a legislature’s RPOV, the question will arise: how should external agents (such as judges) *apply* the legislature’s RPOV to cases?^60^

We propose using principles of *means*–*ends rationality* to guide one agent in applying another’s intention. There is much philosophical debate over what means–ends rationality includes. Broadly, we will assume an agent displays means–ends rationality to the extent that the agent chooses options that have the greatest expected desirability, where ‘desirability’ is determined by all the agent’s other preferences.^61^ Of course, this assumption does not tell you whether to enrol me in a philosophy PhD or merely buy me some books. More is needed in applying agents’ intentions. Several points fill this gap.

First, different items in an agent’s RPOV have differing levels of fundamentality. Items that indicate a ‘purpose’ or ‘goal’ should be taken as more fundamental than other items, by a party who is applying the agent’s RPOV. This is because such purpose-stating items encompass a relatively large portion of the agent’s RPOV, by encompassing or grounding other items. For example, suppose I have stated my purpose is ‘to have conversations with academic philosophers’. When you are applying my intention ‘to be a philosopher’, you should then enrol me in a PhD rather than merely buy me books, given my stated purpose.

Second, parties who are applying others’ intentions might reasonably use their *own* beliefs about the likelihood with which various means will produce the agent’s ends. For example, suppose I intend to be a philosopher, with the purpose of conversing with academic philosophers. When applying this intention, you can reasonably enrol me in a PhD even if you do not know whether I believe a PhD is the means most likely to achieve my end. By inserting your own beliefs about the most likely means to my end, you are remaining true to my purposes, goals and values. This does not descend into constructivism, since judges’ means–ends beliefs have a role only in *applying* legislative intent, not in determining the *content* of that intent.^62^

Third, philosophers have proposed numerous principles to guide our interpretation of other agents. In a seminal article on the subject, the philosopher David Lewis proposed three principles relevant to present purposes: (i) the principle of charity, on which the interpreter should assume their target’s ‘inductive method’ (method for drawing beliefs from evidence) and ‘basic intrinsic values’ roughly match the interpreter’s inductive method and values;^63^ (ii) the rationalisation principle, on which the ‘the beliefs and desires ascribed to [the target] … should be such as to provide good reasons for his behavior’;^64^ and (iii) the principle of generativity, on which the interpreter should assign meanings that are ‘reasonably uniform and simple’.^65^

Importantly, we do not claim that the above will determine a unique correct application in every case. Lewis notes that such principles will leave many indeterminacies—even if the target of interpretation is a human being, and even if the interpreter knows every behaviour their target has ever performed.^66^ Indeterminacies will hardly be decreased if the target is an imperfectly rational legislature.

We make a weaker claim: the RUA provides *guidance* for judicial applications via the methods described in the previous four paragraphs. Moreover, these methods might be only some of the factors in identifying and applying content. Some theories of interpretation might accept these factors while *also* allowing others. As noted in the introduction, we do not advocate a specific theory of interpretation. An intentionalist wishing to use our theory would have to go further than what has been said here and argue that nothing *other than* the RPOV of the legislature should determine legal application. Further, they would, presumably, have to argue for a version of RPOV application that is fairly determinate, although not absolutely determinate (no theory of legal interpretation can guarantee absolute determinacy). The level of determinacy can be tweaked depending on (i) how many of the items (i)–(viii) are included within the legislature’s RPOV and (ii) how one interprets the principles of means–ends rationality and other philosophical principles of interpretation, in particular how such principles should be applied by external agents who are operationalising another agent’s RPOV.^67^

It might be thought that working through the RPOV is rather laborious and places too much of a burden on judges, who have limited time to decide cases. A first comment is that our account is less burdensome than aggregative accounts, which require investigation into the minds of all legislators, and is truer to the facts than common intent accounts, which require judges to posit an unrealistic shared intent. A second comment is that nothing we have listed as part of the RPOV is drastically different from standard considerations that judges examine when interpreting statutes: this will be shown in section 5.

### C. Comparison to Other Accounts

As emphasised, on the RUA, a legislature’s RPOV does *not necessarily* include the political views of all members or the manifestos of all represented political parties, or so on (unless procedures require them to be taken into account). Nor does it include extra-parliamentary items—such as news reports, biographies or legislators’ private notes or diaries. This is important, since common intent or aggregative approaches might consider these as evidence of a legislature’s intention. After all, such items inform us about legislators’ individual thoughts. But such items are of no concern to the legislature’s procedures and structures (whether formal or informal), so they are not taken as evidence for the RUA.

To further see how the RUA differs from aggregative accounts, consider that aggregative accounts determine a legislature’s attitude on proposition (such as the proposition ‘intent *I* is behind law *L*’) by examining members’ attitudes on that proposition. On aggregative accounts, the most prevalent member attitude *just is* the legislature’s attitude. On the RUA, this goes wrong in two places. First, it considers the attitudes of all members. As explained above, procedures and structures might not be sensitive to the attitudes of all members. Second, the aggregative account determines the legislature’s attitude to each proposition individually. The RUA resists considering any proposition in isolation from its role in the legislature’s overall RPOV. This is because the items in any agent’s RPOV are inter-defined.

The RUA also differs from common intent accounts. Like aggregative accounts, proposition-wise common intent accounts presume that the attitudes of members towards a proposition determine the legislature’s attitude towards that proposition. The RUA looks at much else besides members’ attitudes. These include the items—explanatory memoranda, second reading speeches and so on—required by the legislature’s structures and procedures. These need not reflect members’ attitudes (for example, if the items are the result of compromise).^68^

Some common intent models are *not* proposition-wise. In section 2, we mentioned Ekins’s model, on which legislators’ common intent ‘to make law for the common good’ is the ‘standing intention’ behind all laws. On Ekins’s view, the legislature’s intent for specific laws is nonetheless determined by the ‘open proposal’, which is ‘the meaning that a reasonable sole legislator who attends to the context—would be likely to intend’.^69^ The open proposal for a specific statute might not be intended by all legislators. The RUA improves on Ekins’s account in five ways. First, the RUA determines the content of a bill by looking at the other items in the RPOV. Our account thus provides specificity, whereas Ekins’s account is vague on how outsiders (such as judges) might glean the ‘open proposal’. Second, at the application stage, the RUA provides further guidance by appeal to principles of means–ends rationality and other principles of interpretation. Third, the RUA does not unrealistically presume any common intent amongst members—not even common intent to make law for the common good. Fourth, the RUA is not tied to natural law notions of the ‘common good’. Lastly, the RUA explains how the content and interpretation of a statute relates to a broader theory of social entities’ agency.

These comparisons with other accounts might raise an objection: on the RUA, a legislature’s intentions for a given law can be far removed from legislators’ intentions for that law. This might seem to posit a mysterious substance—‘the legislature’—which floats free from individual legislators. Yet the RUA makes no such posit. The procedures, structures and RPOV of a social agent *supervene* entirely on facts about individual humans. Here, we mean ‘supervene’ in a strict sense: x supervenes on y just in case the only way to make a change in x is to make a change in y. The only way to change facts about a legislature is to change facts about individual humans, since the only reason why the procedures and structures of legislatures exist as such are due to individuals.^70^

Further, the RUA is realist rather than constructivist. On the RUA, the legislative intention for a bill is determined in its entirety by the moment the bill is passed. The legislature’s intention is not affected by interpreters, as on the constructivist account. Moreover, the intention behind any given text is static and unchanging on the RUA, unlike on the constructivist view. Our view is, however, somewhat similar to constructivist views: the intent behind any given text to some extent ‘gets away’ from the individual legislators that enact the text. Moreover, we allow principles of means–ends reasoning (and other interpretive principles, such as those from Lewis) to play a role in *applying* a legislature’s intent, as outlined in section 4B. But these principles do not determine the *content* of legislative intent.

### D. Overcoming the Hurdles

It is now possible to explain how the RUA overcomes the four hurdles. First, consider No-Cooperation: legislators do not agree, coordinate or cooperate on the meaning of the text. However, the RUA does not require legislators to agree, coordinate or cooperate; all it requires of them is that they play their roles in the legislature’s structures and procedures, including participating in committees, voting on bills and so on. If they play their roles, then the procedures can be used to produce the mental states of the legislature.

What about Ignorance? Again, the RUA does not require legislators to be aware of the full text of bills or think about what the text means. Their roles in the legislature’s structures and procedures do not require them to be aware of or think about these things. As Hess and Isaacs emphasise, corporate agents primarily require *behaviours* of members, not specific mental states.^71^ As long as enough legislators perform the behaviours required (eg attending committees, voting on bills), this is enough to admit items into a legislature’s RPOV.

The same applies to Insincerity, which stated that the true preferences or intentions of legislators may be hidden from external observers due to legislative compromises and strategic voting. This is no roadblock to applying the RUA: the RUA does not care to look into the minds of legislators to discover their true preferences or intentions.

Finally, the RUA is expressly designed to overcome Grouphood. On the RUA, the legislature’s intent is not just the coincidental actions and intentions of individuals, nor is it just a statistical summary of these. Instead, the legislature’s intent is determined by looking at the other items in the legislature’s RPOV. Those items are admitted into the RPOV by the legislature’s procedures and structures, which are in turn features of the social entity that outlive changes in membership. Therefore, the intention should be attributed to the social entity rather than the individual members.

## 5. Legal Applications

In this section, we demonstrate how the RUA might be applied in its minimal, moderate and broad variants. In what follows, we assume intentionalism is the correct theory of judicial interpretation to show how the RUA *would* be used *if* intentionalism *were* correct. However, we make no commitment about the correct theory of judicial interpretation.

### A. A Minimal Example: Section 51A of Australia’s Migration Act

The Australian Migration Act 1958 (Cth) contains a code of natural justice that has been contested in the High Court several times. Specifically, the Australian government has argued that the code excludes common law rules of natural justice.^72^ Nevertheless, the government’s attempt to exclude the common law has been fraught with difficulties.

In the 2001 case of *Miah*, the Migration Act 1958 (Cth) contained a code of natural justice but did not explicitly state that the code excluded *common law* rules of natural justice.^73^ The relevant division at that time was titled ‘Code of procedure for dealing fairly, efficiently and quickly with visa applications’.^74^ The Australian High Court decided that there was not enough evidence of an intention to remove the common law.^75^

In response, a legislative amendment was passed. The minister’s second reading speech explicitly stated that the intent behind the amendment was to make the statutory code exhaustive:^76^

Therefore, the purpose of this [B]ill is to make it expressly clear that particular codes in the Migration Act do exhaustively state the requirements of the natural justice or procedural fairness hearing rule.

This will have the effect that common law requirements relating to the natural justice or procedural fairness hearing rule are effectively excluded, as was originally intended.

This time the statute read as follows:

**51A Exhaustive statement of natural justice hearing rule**

(1) This Subdivision is taken to be an exhaustive statement of the requirements of the natural justice hearing rule in relation to the matters it deals with.

In addition to section 51A, section 57 of the *Migration Act* at that time restricted the type of information needed to be provided by the minister to *onshore* visa applicants (section 57 was thus a statutory rule of natural justice).^77^ Section 57, which was in the same subdivision as section 51A, did not mention *offshore* applicants. The question was whether section 51A, with section 57, exhausted the rules of natural justice that applied to both *offshore* and *onshore* applicants or whether they only exhausted the rules applying to onshore applicants (since section 57 only mentioned onshore applicants), with common law rules of natural justice applying to offshore applicants.

This amendment was tested in the 2010 case of *Saeed*. The issue was what was meant in section 51A(1) by the subdivision being exhaustive in relation to ‘the matters it deals with’. The High Court suggested there were two options in interpreting ‘matters’. First, the ‘matters’ could be ‘the procedure for dealing with visa applications’,^78^ rendering the Code exhaustive in relation to procedures for dealing with visa applications. The court characterised this as the *single-matter interpretation*, according to which the only source of the Commonwealth’s natural justice obligations to *any* visa applicants, offshore or onshore, is the Migration Act. Second, the ‘matters’ could be the specific subject matters in each individual provision in the subdivision—for example, section 57 dealing with information for onshore applicants.^79^ The court characterised this as the *plural-matter interpretation*, on which the Code was only exhaustive in relation to the matter in section 57, which was onshore applications—leaving the common law to be applied to offshore applicants.

The court adopted the plural-matter interpretation, holding that the common law applied to offshore applicants.^80^ The High Court noted that section 51A used the plural ‘matters’, thus supporting the plural-matter interpretation.^81^ The court said that the phrasing and choice of words—specifically, the plural form—must be assumed to be so for a reason.^82^

Under the minimal approach to the Australian legislature’s RPOV, the RPOV would include the fact that the amendment was the legislature’s response to *Miah*, in addition to the second reading speech and the text of section 51A(1). Given this, the most coherent interpretation of the RPOV excludes the common law for offshore applicants. The single-matter interpretation is a plausible interpretation, for several reasons. First, given the surrounding circumstances, the difference between ‘matters’ and ‘matter’ should not bear the weight of determining the RPOV of the legislature. The minister’s speech states a purpose, so has more sway. Second, the single-matter interpretation could be taken as referring to multiple matters: the ‘matters’ at issue could be understood as the multiple natural justice *obligations* arising from visa applications under the Migration Act. Thus, the fact that ‘matters’ is plural does not support the interpretation that differentiates onshore from offshore applicants. In summary, when the ‘single-matter interpretation’ is combined with other items in the RPOV—specifically, the second reading speech—the most rational interpretation is that common law rules of natural justice were not intended to apply to offshore applicants.

We can also use *Saeed* to differentiate between theories of interpretation. First, consider textualism.^83^ In *Saeed*, the Australian High Court opined, consistently with textualism, that, regardless of explicit clarity of purpose in explanatory memoranda (or, by implication, second reading speeches), there would be no difference to the outcome: ‘Statements as to legislative intent made in explanatory memoranda … *however clear* … cannot overcome the need to carefully consider the words of the statute.’^84^

Justice Heydon further commented: ‘the ultimate question is not what the Parliament intended to do, but what it actually did’.^85^ A textualist would no doubt agree with the High Court that the explanatory memorandum is irrelevant if a strong textual interpretation runs against the memorandum. The textualist would thus agree with the Court in *Saeed*, unlike the RUA-intentionalist.

To separate RUA-intentionalism from an aggregative approach or a proposition-wise common intent approach, consider a variant. Suppose, as above, that in the second reading speech an explicit statement was made that rules of natural justice do not apply to any visa applicants. Further, suppose a significant group of legislators in the governing party were hesitant to approve this. However, suppose a ‘moderate’ coalition of legislators convinced the hesitant coalition to vote for the bill, arguing that the right way to understand the legislation is the plural-matter interpretation. In this scenario, it is possible that the majority will adopt the plural-matter interpretation, making this the aggregated legislative intent. The RUA-intentionalist would disagree: the minister’s speech is relevant, while the internal thoughts of members are not. Alternatively, on a proposition-wise common intent model, suppose the plural-matter interpretation was so widespread that it became the common intent amongst legislators. In this case, the RUA-intentionalist would still reject the plural-matter interpretation. Again, the specific mental states of individual legislators do not form part of a legislature’s RPOV.

### B. Moderate Example: The US Civil Rights Act

In the moderate variant, the legislature’s RPOV includes legislative debates. We illustrate this using Rodriguez and Weingast’s analysis of the legislative history behind Title VII of the US Civil Rights Act.^86^ We depart from the Australian examples and use an American case because there has been a much richer historical investigation of the legislative debates of Title VII (it would go beyond the scope of this article to carry out our own legal historical analysis). In terms of its RPOV, the US Congress does have debates within its standing rules, and in that regard is committed to reasoned legislation.^87^ The debates’ focus was the interaction between sections 703(a) and (h). Section 703(a) makes it unlawful for employers to ‘fail or refuse to hire … or otherwise to discriminate against any individual with respect to his compensation, terms, conditions, or privileges of employment, because of such individual’s race, color, religion, sex or national origin’. Section 703(h) allows for ability tests that measure merits or seniority. At issue was whether sections 703(a) and (h) prohibited indirect discrimination or ‘disparate impact’ regarding employment tests—that is, tests that on their face are not discriminatory but would disproportionately affect people of a certain race. In *Griggs v Duke Power Co*,^88^ the US Supreme Court held that it did. Rodriguez and Weingast claim that the Supreme Court drew the wrong conclusions from the legislative history (we classify them as using an aggregative account).^89^

The court in *Griggs* examined several facts. First, the court inferred from the text of Title VII that one of its purposes was to ‘achieve equality of employment opportunities and remove barriers that have operated in the past to favor an identifiable group of white employees over other employees’.^90^ We assume *arguendo* that the court was correct about this stated purpose. Additionally, and more relevant for the moderate RPOV, the court examined various debates surrounding section 703(h).

When Title VII was introduced, Senators Case and Clark, who supported the bill, issued a memorandum explaining that employers can select based on merits.^91^ Despite this assurance, the more moderate Senator Tower insisted on introducing section 703(h) to allow the use of ability tests.^92^ Proponents of Title VII were initially sceptical of this provision, worrying it would lead to indirect discrimination.^93^ Senator Tower submitted a slightly modified amendment that was accepted when Senator Humphrey (originally opposing the amendment) stated that senators ‘on both sides’ had examined the text and ‘found it to be in accord with the intent and purpose of that title’.^94^ The slight modification, the court itself found, was not substantively different.^95^

The court in *Griggs* stated that, given the overall purpose of removing barriers to employment opportunities, and given Senator Humphrey’s view that section 703(h) did not undermine the bill’s purpose, there was intent that ability tests could not be used in a way that would have disparate impact.^96^ Rodriguez and Weingast take an aggregative approach, arguing the Court made the wrong decision. According to them, in cases where compromise is needed to pass a bill, statements of ardent supporters (like Senators Case, Clark and Humphrey) cannot be trusted because they are attempting to sell the bill both to ardent supporters and to those who have not been fully convinced.^97^ It is the moderate, *pivotal*, legislators (like Tower) that matter.^98^ When Senator Humphrey assented to section 703(h), this indicated that the majority had allowed the pivotal senators (like Tower) to include their coalition’s policy in the bill (by voting to let it in).^99^ Otherwise, the entire legislation was at risk of collapsing.^100^ Rodriguez and Weingast provide evidence that Tower’s reason for introducing the amendment was to prevent Title VII from being interpreted similarly to state laws, where indirect discrimination was recently found to be prohibited.^101^ Because aggregative theorists are concerned with what a majority of legislators were thinking, the Supreme Court’s interpretation was incorrect.

The moderate RUA sides with the Supreme Court over Rodriguez and Weingast. On this approach, debate contributions are included in the legislature’s RPOV, at least when doing so does not introduce rampant contradiction. The Supreme Court identified *some* debate contributions that indicated the unqualified use of employment tests (Senator Tower’s statements). But, given Senator Humphrey’s statement that section 703(h) would not undermine the intent and purpose of Title VII *and* given the court’s reading of the Title’s purpose, the more rationally coherent application of the RPOV is a prohibition of indirection discrimination. When the debate contributions contradict each other, at the stage of application we should privilege those contributions that accord with the statute’s *purpose* (as explained in section 4B on means–ends rationality): in this case, addressing racial discrimination. What the majority of legislators thought—perhaps represented by the pivotal legislators like Tower—does not matter, since the items which are fundamental to the RPOV do not depend on the *number* of individuals supporting them.

### C. Broad Example: Australia’s Principle of Legality

Interpretive canons are principles that instruct judges on how to interpret certain provisions. For example, the *principle of legality* states that where a statute potentially infringes on common law rights, it should be assumed the legislature does not intend to cancel such rights unless there is evidence otherwise.^102^ Some canons are laid out in statute. These are straightforwardly included within the *minimal* version of a legislature’s RPOV. Other canons are a matter of common law. These enter the legislature’s RPOV only on the *broad* version. Such canons are known by legal practitioners. Therefore, in the legislative context, the canons might be taken as falling under ‘contextual general knowledge’ as characterised in section 4A. For example, in the Australian case of *Electrolux*, Gleeson CJ opined that the principle of legality is ‘a working hypothesis, the existence of which *is known both to Parliament and the courts*, upon which statutory language will be interpreted’.^103^

If interpretive canons are included in the RPOV, they are still secondary to (i) the items that explain the purpose of bills and (ii) items that are more directly sourced from procedures and structures. The secondary status of canons follows from the means–ends rationality approach to applying legislative intent: purposes or goals trump general knowledge. Additionally, the legislature’s procedures and structures orient the legislature’s RPOV towards rational coherence, so items that enter more directly, via the procedures and structures, should take priority over those that arise through a more diffuse ‘general knowledge’ route.

As an example, consider the Australian case of *Probuild Constructions*. Here, the common law right to judicial review was taken to be trumped by statute.^104^ The relevant statute, the Building and Construction Industry Security of Payment Act 1999 (NSW) (SoPA), established a scheme by which contractors would be able to get progress payments as they continued construction work (rather than only being paid once all the work was done). The court cited the second reading speech of the minister, which stated that the statute was meant to ‘stamp out the practice of developers and contractors delaying payment to subcontractors and suppliers’ and achieve a ‘unique form of adjudication of disputes’.^105^ Further, the court noted that the subject matter of SoPA was not finality or conclusive determination of the law. The minister stated that ‘Cashflow is the lifeblood of the construction industry’.^106^ The minister also noted that the omission of a statutory right of appeal was deliberate.^107^ Taking these items together, the court concluded that it would undermine the purpose of the act if every interim decision of an adjudicatory could be appealed to a court.^108^

The intent of the NSW legislature—as determined by the minister’s statements, which are included in the minimal RPOV—was to ensure contractors and suppliers were paid as construction went along. They had other deliberate ‘means’ items to allow for quick adjudication and no statutory appeal. Additionally, under the broad RPOV, the legislature was committed to the interpretive canon of the principle of legality, which allows for a common law appeal to the courts. However, given the other items in the RPOV—particularly the intent articulated by the minister—the plausibility of upholding the principle of legality is weak. This case, therefore, demonstrates that the broad RPOV does not give judges undue influence over the content of legislative intent.

Existing realist theories of legislative intent have problems supporting interpretive canons like the principle of legality. Most legislators do not know about these canons or are unlikely to have thought along the lines presupposed by them.^109^ However, on the broad RUA, the inclusion of the canons does not depend on individual legislator mental states. Instead, it is justified if and when it is the kind of general knowledge necessary for reasonable law making. The broad RPOV, therefore, better explains why interpretive canons might be legitimate presumptions of legislative intent.

## 6. Conclusion

The debate about legislative intent has largely proceeded without recourse to philosophical debates about the agency of social entities.^110^ When those debates are examined and synthesised, we find that what matters most is not the aggregated intentions of members, or even the commonly held intentions or other attitudes of members. Rather, what matters are the procedures and structures that admit any given attitude or commitment into the bundle of attitudes and commitments to which the social entity itself is committed. Those procedures and structures do not require any particular mental states from members. As we explained, this ‘rational unity account’ of agency can fruitfully be used to develop an account of legislative intent. The account overcomes the four hurdles that, we argued, hamper existing realist accounts of legislative intent: the obstacles of No-Cooperation, Ignorance, Insincerity and Grouphood. Furthermore, we demonstrated how different results can occur depending on what theory one endorses.

That said, we have not claimed that the rational unity account will always answer every question one might have about statutory interpretation. Sometimes, crucial terms in a statute will remain vague or underspecified, even after all items in the legislature’s RPOV have been considered. We have not claimed that there is *always* a legislative intent that goes beyond what is explicitly stated in the text, or that there can never be reasonable debate about application. We have instead aimed to demonstrate how interpretation and application is possible in a way that improves upon the existing aggregative and common intent accounts.

## Footnotes

1 Richard Ekins, *The Nature of Legislative Intent* (OUP 2012) 103.

2 As s 2 explains, some aspects of the account are sympathetic to Richard Ekins’s common intent account.

3 Ekins (n 1) 23, 115, 239 makes a similar distinction. However, our view will allow the legislature’s intentions and commitments to depart more strongly from the legislators’ intentions and commitments than Ekins allows. Ekins explains legislative agency with recourse to legislators’ intentions: Ekins (n 1) 52.

4 John Manning, ‘Inside Congress’ Mind’ (2015) 115 (7) Colum L Rev 1911; Kenneth Shepsle, ‘Congress Is a “They”, Not an “It”: Legislative Intent as Oxymoron’ (1992) 12 International Review of Law and Economics 239; Ronald Dworkin, *Law’s Empire* (Belknap 1986) 313–54; John Gardner, ‘Some Types of Law’ in D Edlin (ed), *Common Law Theory* (CUP 2007) 51.

5 Trevor Allan, ‘Legislative Supremacy and Legislative Intent: A Reply to Professor Craig’ (2004) 24(4) OJLS 563.

6 Ekins (n 1); David Tan, ‘Uncommon Legislative Attitudes: Why a Theory of Legislative Intent Needs Nontrivial Aggregation’ (2021) 34(2) Ratio Juris 139.

7 Shepsle (n 4) 239.

8 TRS Allan, who has explicitly adopted the term ‘constructive legislative intent’, invokes an imaginary dialogue: ‘there is no sharp line between genuine interpretation … and “pretended” interpretation … there is only our imaginary dialogue, conducted in good faith with draftsmen and legislators whose commitments to constitutional justice … we must broadly take to match our own … [a]n element of creativity is inherent in the task of adapting the text to the insistent demands of unfolding events’. Trevor Allan, ‘Constitutional Dialogue and the Justification of Judicial Review’ (2003) 23 (4) OJLS 563, 572. Allan’s view is somewhat vague. We sympathise with Craig’s worries: ‘The concept [of constructed legislative intent] is repeatedly said to be a ‘metaphor’, although a metaphor for what is never made apparent’. Paul Craig, ‘Legislative Intent and Legislative Supremacy: A Reply to Professor Allan’ (2004) 42(4) OJLS 585, 585.

9 Ekins (n 1); Richard Ekins and Jeffrey Goldsworthy, ‘The Reality and Indispensability of Legislative Intention’ (2014) 36 Syd LR 39; Neil Duxbury, *Elements of Legislation* (CUP 2012) 106–19.

10 Tan, ‘Uncommon Legislative Attitudes’ (n 6); David Tan, ‘Defending Aggregated Legislative Intent’ (forthcoming) CJLJ; Daniel Farber and Phillip Frickey, ‘Legislative Intent and Public Choice’ (1988) 74(2) Va L Rev 423. Ronald Dworkin at times approaches an aggregative view of the legislature (R Dworkin, ‘Social Sciences and Constitutional Rights—the Consequences of Uncertainty’ (1977) 6 Journal of Law and Education 3, 10), though he ultimately denies legislative intent: Dworkin, *Law’s Empire* (n 4). See Ekins’s insightful discussion of Dworkin in Ekins (n 1) 77–85.

11 Ronald Dworkin, *Taking Rights Seriously* (Duckworth 1977) 85; Andrei Marmor, ‘The Pragmatics of Legal Language’ (2008) 24(4) Ratio Juris 423.

12 Jamie Blaker, ‘Is Intentionalist Theory Indispensable to Statutory Interpretation?’ (2017) 43(1) Mon LR 238.

13 Shepsle (n 4); John Manning, ‘Second-Generation Textualism’ (2010) 98 CLR 1287.

14 Ekins (n 1) 23.

15 Kenneth Arrow, *Social Choice and Individual Values* (Wiley 1951).

16 Christian List and Phillip Pettit, *Group Agency: The Possibility, Design, and Status of Corporate Agents* (OUP 2011) ch 2; George Mackie, *Democracy Defended* (CUP 2003).

17 Farber and Frickey (n 10); Arthur Lupia and Mathew McCubbins, ‘Lost in Translation: Social Choice Theory is Misapplied Against Legislative Intent’ (2005)14 Journal of Contemporary Legal Issues 385.

18 Christian List, ‘Three Kinds of Collective Attitudes’ (2014) 79(S9) Erkenntnis 1601.

19 There are versions of common intent that require more than mere unanimity, such as the ‘shared intentions’ theorised by Michael Bratman, *Shared Agency* (OUP 2014). Perhaps such bodies bear group-level agency, as theorised in s 3 (for an argument that they do not, see Stephanie Collins, *Group Duties* (OUP 2019) ch 2). The closer such groups come to bearing agency, and thus achieving Grouphood, the further they will be from real-world legislatures, because the more harmony there will be amongst members.

20 Ekins (n 1) 234.

21 ibid 236.

22 Ekins argues that the *central case* of the legislature is one in which all legislators have a unanimous ‘standing intention’ to ‘debate in a highly structured fashion so as to create policies *for the common good*’ (ibid 220, emphasis added), even though real-world legislators depart from this central case. However, as others have observed, if real-world legislatures depart from this central case, then it is unclear how this analysis captures real-world legislative intent. See Francesco Ferraro and Adriano Zambon, ‘Ekins’s Moral Assumptions and their Impact of the Analysis of Legislation’ (2019) 64(1) Am J Juris 19, 20–24; Andrea Dolcetti, ‘The Central Case Method in The Nature of Legislative Intent’ (2019) 64(1) Am J Juris 41, 54, 64.

23 Ekins seems to derive his account from some version of Finnis’s central case method, but that is an account of social facts and not intentions.

24 We will use Peter French, *Collective and Corporate Responsibility* (Columbia UP 1984); Carol Rovane, *The Bounds of Agency* (OUP 1998); List and Pettit (n 16); Tracy Isaacs, *Moral Responsibility in Collective Contexts* (OUP 2011); Raimo Tuomela, *Social Ontology: Collective Intentionality and Group Agents* (OUP 2013); Deborah Tollefsen, *Group as Agents* (Polity 2015); Kendy Hess, ‘Does the Machine Need a Ghost? Corporate Agents as Nonconscious Kantian Moral Agents’ (2018) 4(1) Journal of the American Philosophical Association 67. For a similar account of organisational agency, see Stephanie Collins, *Organizations as Wrongdoers: from Ontology to Morality* (OUP 2023) ch 1.

25 Donald Davidson, *Essays on Actions and Events* (OUP 1980).

26 Functionalism is endorsed in social ontology by List and Pettit (n 16); Bryce Huebner, *Macrocognition* (OUP 2014); Brian Epstein, *The Ant Trap* (OUP 2015). Others do not explicitly endorse functionalism, but are well understood in these terms (eg French (n 24); Isaacs (n 24)).

27 As Donald Davidson famously put it, attitudes towards propositions come as a ‘matched set’, in which ‘to have one is to have a large complement’. Donald Davidson, *Subjective, Intersubjective, Objective*, Philosophical Essays vol 3 (OUP 2001) 96. David Lewis likewise took an agent’s whole set of propositional attitudes and meanings to be interpretable only in an inter-dependent way: D Lewis, ‘Radical Interpretation’ (1974) 23 Synthese 331.

28 Rovane (n 24); Tollefsen (n 24) and Hess (n 24) also employ this concept.

29 One need not be a functionalist to accept the RUA. Hess outlines interpretationist, dispositionalist and representationalist approaches to the agency of social entities, all of which employ the core concept of an RPOV and all of which are consistent with the coming arguments. See Kendy Hess, ‘Because They Can: The Basis for the Moral Obligations of (Certain) Collectives’ (2014) XXXVIII Midwest Studies in Philosophy 203, 211–13. We have outlined functionalism simply to provide the reader with one robust and popular theory of mind, which supports the arguments to come. The reader could instead use interpretationist, dispositionalist or representationalist theories of mind to support those arguments. On combining functionalism and interpretationism in theorising group agency, see Collins (n 24).

30 Rovane (n 24) 86. As Pettit puts it, a rational collective agent strives ‘to endorse only views that can be integrated with one another in a single rational vision’: Philip Pettit, ‘Akrasia, Collective and Individual’ in Christine Tappolet and Sarah Stroud (eds), *Weakness of Will and Practical Rationality* (Clarendon Press 2003) 79).

31 To see why, consider thought experiments—dating back to John Locke and depicted in films such as *Freaky Friday*—in which two humans swap RPOVs. Locke’s example is a prince and a cobbler who suddenly swap all memories, beliefs, preferences, etc: J Locke, *An Essay Concerning Human Understanding* (Peter H Nidditch ed, OUP 1975). The prince is trapped in the cobbler’s body, and vice versa, which implies that agents should be associated with an RPOV, not an animal body or brain. As Rovane (n 24) has argued, this opens the door to social agents that have an RPOV but lack one animal body or brain, such as (we will argue) the legislature.

32 For example, Shell acts in a coherent way to maximise profits by drilling for oil, in accordance with a wide range of group-level beliefs, preferences, hopes, fears and so on. Importantly, an RPOV can be viewed as a ‘psychological economy’ (Rovane (n 24) 21), a diverse smorgasbord of beliefs, preferences and so on, which reflect various competing considerations, where give-and-take occurs between the different parts of the economy. An RPOV, then, is not a static, harmonious or well-orchestrated tableau of mental states and action dispositions; instead, it is a dynamic, diverse and sometimes contradictory bundle, in which full coherence is an ideal rarely achieved (Rovane (n 24) 22).

33 We use ‘social entity’ to be neutral on the vexed question of whether social entities are groups, sets, pluralities, fusions, abstracta and so on. We pick out the category ‘social entities’ by its paradigm examples, listed in the main text.

34 Peter French, ‘The Corporation as a Moral Person’ (1979) 16 American Philosophical Quarterly 207, 212; French, ‘Collective and Corporate Responsibility’ (n 24).

35 French, ‘Collective and Corporate Responsibility’ (n 24) 41.

36 As mentioned above (n 33), we do not assert that the legislature is a group, that is, that it is composed entirely of people. List and Pettit’s theory can be applied to legislatures even if legislatures are abstracta, for example.

37 List and Pettit (n 16) 42–58.

38 ibid 54–8.

39 Tuomela (n 24) 21.

40 Kendy Hess, ‘The Peculiar Unity of Corporate Agents’ in K Hess, V Igneski and T Isaacs (eds), *Collectivity: Ontology, Ethics, and Social Justice* (Rowman & Littlefield 2018) 35–60.

41 Kendy Hess, ‘The Modern Corporation as Moral Agent: The Capacity for “Thought” and a “First-Person Perspective”’ (2010) 26 Southwest Philosophy Review 61.

42 Hess, ‘Does the Machine Need a Ghost?’ (n 24).

43 Tollefsen (n 24) 104.

44 ibid 109.

45 Isaacs (n 24) 28.

46 ibid 29.

47 This differs from Ekins’s vision of the legislature in three important ways. First, Ekins does not ultimately ground his theory in the legislature’s procedures and structures (indeed, he says procedures are not necessary for group action: Ekins (n 1) 61. Instead, he focuses on goals held in common by *the legislators*. Second, Ekins believes all legislators are committed to the goal of making laws *for the common good.* We think this is an illicit idealisation. Third, Ekins considers the intent for each law *in isolation* from other laws: Ekins (n 1) 13, 25, 49, 52–3, 64, 125, 127, 141. On our account, the entire body of legislation, and more besides, contributes to the intent behind each law, since (we will argue) the legislature’s procedures and structures properly include all these items in the legislature’s RPOV.

48 Department of House of Representatives, *Standing Orders* (2 August 2022) Ord 141; Australian Senate, *Standing Orders* (July 2021) Ord 112 (in the Senate, no explanatory memorandum is required).

49 Department of House of Representatives, *Standing Orders* (2 August 2022) Ord 142; Australian Senate, *Standing Orders* (July 2021) Ord 114.

50 Department of House of Representatives, *Standing Orders* (2 August 2022) Ords 148–51; Australian Senate, *Standing Orders* (July 2021) Ords 115–18 (although this is not called ‘Consideration-in-detail’ in the Senate).

51 Department of House of Representatives, *Standing Orders* (2 August 2022) Ord 155; Australian Senate, *Standing Orders* (July 2021) Ord 122.

52 Some scholars propose that legislative debates are just ‘cheap talk’. See Cass Sunstein, ‘Interpreting Statutes in the Regulatory State’ (1989) 103 Harv L Rev 407, 428–9. For a response to this, see McNollgast, ‘Legislative Intent: The Use of Positive Political Theory in Statutory Interpretation’ (1994) 57(1) LCP 1, 25–9. Our account takes no stand on this.

53 Ekins likewise states that ‘Reflection on the standing orders that structure the House’s deliberation and decision confirms … that its standing intention is to form and enact complex, reasoned, coherent proposals, which set out and constitute its particular intention in any legislative act’ (Ekins (n 1) 224). However, he infers the *content* of these proposals from open proposals, which we defined in s 2 as deriving from the intentions of a hypothetical and idealised ‘sole legislator’ (Ekins (n 1) 231). The RUA makes no such reference to hypotheticals.

54 Furthermore, consider how the legislature relates to other social entities. Each of Australia’s largest political parties has a constitution stipulating that parliamentary members are to implement policies consistent with their party’s objectives: Australian Labor Party, *National Constitution* (18 December 2018) Cls 4, 14(d); Liberal Party of Australia, *Federal Constitution* (2017) Cls 2, 9.3, 15.1, 15.2. And the Office of Parliamentary Counsel (which drafts the bills) requires policy instructions from the relevant agencies who want to introduce bills. Office of Parliamentary Counsel, *OPC’s Drafting Services: A Guide for Clients* (7th edn, July 2022) 9. Thus, these other social entities impute the possibility of a legislative RPOV. Insofar as these entities’ advice is incorporated into the text or explanatory memorandum of a bill, their views enter the legislature’s RPOV—but only because their advice has been incorporated via the legislature’s procedures and structures.

55 One might worry that explanatory memoranda, which come from the *executive*, should not be able to determine *legislative* intent. However, the legislature can coherently take as important the intent of another agent (the executive), treating it as an external advisor. Additionally, there is nothing in our theory that states that the explanatory memorandum *will always* dominate the other items in the RPOV. Lastly, even when the explanatory memorandum dominates the other items in the RPOV, this is simply consistent with the reality of Westminster systems, in which the leader of the executive, the Prime Minister, is also the leader of the majority party in the legislature.

56 To see this, suppose a commander at *t*_1_ instructs their division to do *X* and at *t*_2_ instructs *not-X*, intending both to be followed. The correct interpretation, assuming reasonableness, is that the commander either wanted the most recent command to trump the earlier one or wanted both commands to be interpreted in a way that is consistent. We should assume the commander reneges the earlier instruction *only if* the two commands *cannot* be rendered consistent. The same goes for legislatures’ commitments to prior statutes.

57 In Australia, for the doctrines of ‘harmonious construction’ and ‘implied repeal’, see *Commissioner of Police (NSW) v Eaton* (2013) 252 CLR 1 and *Saraswati v The Queen* (1991) 172 CLR 1, respectively. The idea of harmonious construction is, of course, not straightforward. The general idea is to find interpretations of seemingly inconsistent provisions that can make sense of all the words and clauses of those provisions (*Eaton* 33[98]). If there is only one such interpretation, then the solution has been provided. If there are multiple such interpretations, then our suggestions regarding applying legal content in s 4B below would be relevant. (To foreshadow: we will propose means–ends reasoning, among other principles, to find the interpretation that best satisfies the overall commitments of the legislature in those areas of law.) Now of course it might be that no interpretation can make sense of the conflicting provisions, and as such implied repeal would be the appropriate doctrine to fall back on: the most recent provision trumps the older one.

58 Even if one rejects contextual general knowledge as being a *constitutive part* of the legislature’s RPOV, contextual general knowledge might still influence the *application* of the legislature’s RPOV, as discussed in s 4B.

59 This distinction has been considered by originalist scholars under the notion of *interpretation* (content identification) and *construction* (application): Lawrence Solum, ‘The Interpretation–Construction Distinction’ (2010) 27 Constitutional Commentary 95; Keith Whittington, *Constitutional Construction: Divided Powers and Constitutional Meaning* (Harvard UP 1999); Randy Barnett, ‘Interpretation and Construction’ (2011) 34 Harv JL & Pub Pol’y 65. We use ‘interpretation’ to refer to *both* content identification *and* application of that content. On the distinction statutory interpretation, consider Dale Smith, ‘What Is Statutory Purpose?’ in Lisa Burton Crawford, Patrick Emerton and Dale Smith (eds), *Law Under a Democratic Constitution: Essays in Honour of Jeffrey Goldsworthy* (Hart Publishing 2019).

60 Before we answer, note that our account has already gone beyond other accounts in answering this question, since, on our view, intentionalist legal applications must be sensitive to all the items within a legislature’s RPOV—while ignoring legislators’ (aggregated or common) views on the statute in question.

61 One might think this assumption describes a ‘reasonable’ agent rather than a merely ‘rational’ one. We take no stand on this; our point is that this means–ends assumption can be made of the legislature. This is because the legislature is an agent with an RPOV that is committed to reasoned legislation, as explained earlier.

62 If contextual general knowledge was rejected from the legislature’s RPOV (see n 58), it would appear at this stage of the adjudicating process when *applying* legislative intent, since contextual general knowledge might be the basis of judges’ means–ends beliefs.

63 Lewis (n 27) 336–7. For judges, the ‘basic intrinsic values’ might include such things as constitutionally enshrined rights.

64 ibid 337. ‘Good reasons’ might refer to the judge’s own view on what would be a ‘good reason’ for the legislature’s behaviour of passing a particular law. Again, though, the judge’s perspective arises only when *applying* legislative intent, not determining the *content* of that intent, as under constructivism. Further, the judge’s view on ‘good reasons’ should be utilised only when all above-mentioned methods are exhausted.

65 ibid 339.

66 ibid 343.

67 As an anonymous reviewer noted, some relevant items in the RPOV (such as committee reports on a specific bill) might be mutually contradictory. In general, such contradictions should be resolved by looking to the *entirety* of the legislature’s RPOV. The statute should be interpreted in a way that fits best with the rest of the RPOV taken as a whole, where this includes (for example) ministers’ past speeches on other related bills. Where such sources of determinacy run out, we must accept that the legislature’s intent is indeterminate—much as my above-mentioned intention ‘to be a philosopher’ is indeterminate.

68 One might think common intent nonetheless underpins our account: one might mistakenly think that a legislature’s commitment to rational unity amounts to a common commitment amongst members to the legislature’s rational unity. However, the RUA does not assume any unanimity amongst legislators, not even a unanimous intent to ‘produce legislation that has some intention or other’ or ‘make the legislature rationally unified’. Such an assumption would be an illicit idealisation of real-world legislators. Moreover, the assumption is simply not needed: the legislature’s commitment to rational unity is not a commitment that inheres in being held in common by members. Rather, the legislature’s commitment to rational unity (and to the content of its RPOV) is encoded in the procedures and structures that govern the legislature.

69 Ekins (n 1) 236.

70 However, the objection does get something correct: the relevant kind of supervenience is ‘global’ rather than ‘local’. The distinction between ‘local’ and ‘global’ supervenience is common in the philosophy of mind; see Jaegwon Kim, *Supervenience and Mind: Selected Philosophical Essays* (CUP 1993). By this, we mean that a legislature’s intent for law *L* is determined by facts about individuals *other than* facts about legislators’ intents for *L*. If the legislature’s intent for *L* supervened *only* on the facts about legislators’ intent for *L*, then *L*’s intent would supervene on ‘local’ facts. Instead, under the RUA, *L*’s intent is determined by a range of *facts other than* legislators’ intents for *L*. These facts are the other items in the legislature’s RPOV. These items depend on individuals—but they do not necessarily depend on *legislators’ intentions for L*. Thus, we agree with the criticisms of Ekins given in Tan, ‘Uncommon Legislative Attitudes’ (n 6) 146ff, which arise because Ekins views legislative intentions as supervening only on relatively ‘local’ facts. Instead, the items might result from bargaining, compromise, consultation and so on. Such origins do not undermine their status as items in the legislature’s RPOV.

71 Hess, ‘Does the Machine Need a Ghost?’ (n 24); Hess ‘The Modern Corporation as Moral Agent’ (n 41); Isaacs (n 24).

72
*Re Minister for Immigration and Multicultural Affairs; Ex Parte Miah* (2001) 206 CLR 57; *Saeed v Minister for Immigration and Citizenship* (2010) 241 CLR 252.

73
*Ex Parte Miah* (n 72) 70[34].

74 ibid 69[32], 83[88].

75 ibid 87–8.

76
*Saeed* (n 72) 264[30]–[31].

77 ibid 259–60[16], 261[21].

78 ibid 266[36].

79 ibid 266–7[38]–[39], 274[66].

80 ibid 264–7.

81 ibid 266[38].

82 ibid 267[39].

83 eg Manning, ‘Second-Generation Textualism’ (n 13); Antonin Scalia, *A Matter of Interpretation: Federal Courts and the Law* (Princeton UP 1997) ch 1; Jeremy Waldron, *Law and Disagreement* (OUP 1999) 143.

84
*Saeed* (n 72) 264–5[31] (emphasis added).

85 ibid 277–8[74].

86 Daniel Rodriguez and Barry Weingast, ‘The Positive Political Theory of Legislative History: New Perspectives on the 1964 Civil Rights Act and Its Interpretation’ (2003) 151(4) U Pa L Rev 1417.

87 Rules of the House of Representatives for the One Hundred and Seventeenth Congress (2 February 2021) Rules XVII—XVIII; Standing Rules of the Senate (24 January 2013) Rule XIX.

88 401 US 424 (1971).

89 Rodriguez and Weingast (n 86) 1501–10.

90
*Griggs* (n 88) 429–30.

91 ibid 434.

92 ibid 435.

93 ibid.

94 ibid 435–6.

95 ibid 436, fn 12.

96 ibid 436.

97 Rodriguez and Weingast (n 86) 1439, 1444, 1507.

98 ibid 1439, 1444.

99 ibid 1506.

100 ibid 1463.

101 ibid 1504–5.

102
*Coco v The Queen* (1994) 179 CLR 427.

103
*Electrolux Home Products Pty Ltd v Australian Workers’ Union* (2004) 221 CLR 309, 329[21] (emphasis added).

104
*Probuild Constructions (Aust) Pty Ltd v Shade Systems Pty Ltd* (2018) 264 CLR 1. The appellants had characterised their arguments as depending on the principle of legality and Edelman J agreed that it could be characterised as such, although his honour ultimately rejected the argument (34–5). The characterisation of this case as a rebuttal of the principle of legality is also considered in Dan Meagher, ‘On the Wane? The Principle of Legality in the High Court of Australia’ (2021) 32 PLR 61.

105
*Probuild* (n 104) 15[36].

106 ibid 17[40].

107 ibid 18[43]

108 ibid 19[48].

109 Brendan Lim, ‘The Normativity of the Principle of Legality’ (2013) 37(2) MULR 372; Dale Smith, ‘Is the High Court Mistaken About the Aim of Statutory Interpretation?’ (2016) 44(2) FL Rev 227.

110 One exception is Ekins (n 1); our asides have outlined the limitations of his account.

